# A High-Entropy True Random Number Generator with Keccak Conditioning for FPGA

**DOI:** 10.3390/s25061678

**Published:** 2025-03-08

**Authors:** Valeria Piscopo, Alessandra Dolmeta, Mattia Mirigaldi, Maurizio Martina, Guido Masera

**Affiliations:** Department of Electronics and Telecommunications, Politecnico di Torino, 10129 Torino, Italy; alessandra.dolmeta@polito.it (A.D.); mattia.mirigaldi@polito.it (M.M.); maurizio.martina@polito.it (M.M.); guido.masera@polito.it (G.M.)

**Keywords:** True Random Number Generators, ring oscillators, entropy, open-source hardware, key generation, FPGA

## Abstract

Any cryptographic system strongly relies on randomness to ensure robust encryption and masking methods. True Random Number Generators play a fundamental role in this context. The National Institute of Standards and Technology (NIST) and the Bundesamt für Sicherheit in der Informationstechnik (BSI) provide guidelines for designing reliable entropy sources to fuel cryptographic Random Bit Generators. This work presents a highly parameterized, open-source implementation of a TRNG based on ring oscillators, complemented by an optimized Keccak conditioning unit. The design process is accompanied by a thorough study of the relevant literature and standards, specifying the requirements for reliable entropy sources in cryptographic systems. The design of the TRNG proposed in this paper aims to strike a balance between area, throughput, power consumption, and entropy, while adhering to these guidelines. The proposed design has undergone extensive testing and validation and has successfully passed the NIST SP 800-22, NIST SP 800-90B, and BSI AIS-31 tests, achieving a min-entropy per bit of 0.9982 (NIST) and 0.9998 (BSI).

## 1. Introduction

Protecting sensitive information through cryptography is a fundamental requirement of the modern digital infrastructure, playing a vital role in a wide variety of fields, e.g., communications, finance, healthcare, and national security. Ensuring data confidentiality, integrity, and authenticity is dependent on the generation of secure encryption keys and other cryptographic primitives, which are the fundamental building blocks of robust security frameworks. Randomness is a key element of these frameworks, as it plays a critical role in generating secure encryption keys and preventing cryptographic attacks, but ensuring the secure implementation of cryptographic algorithms is equally critical. Over the past few years, a significant threat has arisen in the form of side-channel attacks. These attacks exploit unintended information leakage from cryptographic systems—variations in power usage, execution time, electromagnetic signals—to uncover sensitive data and undermine security. Randomness plays a dual role here: it is indispensable not only for generating secure encryption keys but also as a foundation for countermeasures like masking, which protect implementations by concealing intermediate computations and minimizing information leakage [1,2,3].

The generation of randomness is a pillar of cryptographic systems, providing the unpredictability required for various security mechanisms. There are two primary types of randomness sources: Pseudo Random Number Generators (PRNGs) and True Random Number Generators (TRNGs). PRNGs, while widely used, rely on deterministic algorithms to produce sequences of numbers from a fixed seed. This predictability makes them vulnerable to certain attacks if the seed or algorithm is compromised. In contrast, TRNGs offer a higher degree of security by exploiting the inherent unpredictability of physical phenomena, ensuring greater resilience against attacks that target the generation process.

In digital circuits, jitter events caused by semiconductor noise, power supply fluctuations, temperature variations, or cross-talk are commonly exploited as a source of randomness. Table 1 summarizes the most commonly used digital noise sources in the literature. TRNG architectures often employ Ring Oscillators (ROs) [4,5,6] and Phase Locked Loops (PLLs) [7,8,9] to generate clock signals affected by jitter. RO-based entropy sources demonstrate high versatility for implementation in both ASIC and FPGA, supported by a well-established behavioral model and entropy justification [4,10]. Although they remain susceptible to frequency injection attacks, this vulnerability can be mitigated through voltage regulation [11,12]. Improving the throughput of the noise source and/or ensuring the bitstream uniformity may require an additional layer of post-processing. To this end, the NIST [13,14] and BSI [15] guidelines allow the integration of an optional conditioning component that implements a deterministic function.

**Contribution**. This paper presents a comprehensive TRNG architecture based on an RO noise source, including a hardware implementation of the Keccak hash function [19]. This component can be used as a conditioning block as well as a Deterministic Random Bit Generator (DRBG). The architecture is highly configurable, accommodating varying power, performance, and security requirements. The TRNG has been implemented on a Xilinx Artix 7 FPGA and has undergone rigorous validation through NIST and BSI test suites, providing optimal min-entropy results and excellent performance. Furthermore, all the requisite resources are accessible as open-source material on GitHub (https://github.com/vlsi-lab/TRNG, accessed on 5 March 2025).

**Organization**. The article is structured as follows. Section 2 provides an overview of the state-of-the-art in RO-based noise sources and TRNG architectures. Section 3 reviews the NIST and BSI methodologies to test the randomness and assess the entropy of data. Section 4 focuses on the RO-based noise source, presenting an analysis of the impact of the design parameters on area, power, and randomness. Section 5 details the complete proposed architecture, including the Keccak conditioning component. The results of the proposed TRNG are presented in Section 6 and then compared with those of recent works. Finally, the concluding Section 7 summarizes the solution and possible future works.

## 2. State-of-the-Art

This section aims to provide a review of some of the most relevant noise sources (Section 2.1) and TRNG architectures (Section 2.2) presented in the literature. Since our design is based on an RO noise source, only similar architectures will be considered.

### 2.1. RO-Based Noise Sources

The selection of a true random source has a major impact on the performance of the TRNG, so most of the work in the literature focuses primarily on this component. Sunar et al. [4] were the first to propose a design for an RO-based noise source. In their model, the outputs of *n* parallel ROs are XORed and then sampled. This work serves as a foundational starting point for various RO-based noise sources. An enhanced version of this solution is presented in [20]: an additional sampler (D Flip-Flop) at the end of each parallel RO reduces the setup and hold-time constraints of the sampling period. Lu et al. [5] improve the TRNG randomness by employing an additional RO as a multiphase sampler, which samples the ROs at different time intervals. The design is tested under different temperatures and supply voltages, but the NIST SP 800-22 [21] and NIST SP 800-90B [14] tests are not conducted in accordance with the original NIST specifications. For instance, only 30 sequences are tested with the NIST SP 800-22 suite, even though α=0.01. In [22], the authors propose a modified Ring Oscillator (RO) noise source by employing a configurable Transition Effect Ring Oscillator (TERO). The TERO’s stochastic model is validated on a real FPGA implementation, yielding a final per-bit entropy of 0.9993, as confirmed by BSI AIS-31 tests [15]. Della Sala et al. [23] propose a Latched Ring Oscillator (LRO) noise source to obtain a compact and high-entropy TRNG, albeit with reduced throughput. The design is validated under different environmental conditions with NIST SP 800-22 and BSI AIS-31 tests. A Dual-Ring Oscillator (DRO) is the core component of the design presented in [24]. Noise sources based on 2-bit DROs and 8-bit DROs are compared to evaluate the trade-off between area occupation and throughput. In addition to NIST SP 800-22 tests, the NIST SP 800-90B suite is applied, yielding a non-IID assessment and a min-entropy of 0.995 for the 2-bit version. As concerns the BSI AIS-31 suite, only the *T8* test is applied. The architecture demonstrates resilience against both PVT attacks and frequency injection attacks up to 300 MHz. Zhang et al. [25] present an RO-based dynamic hybrid entropy source. The design’s base cell leverages jitter extraction and metastability. Coupling and feedback strategies are employed to further enhance the output randomness without resulting in significant area and power overhead. The architecture provides a low area, high throughput solution, with a power consumption of 0.068 W, and it is resilient to voltage and temperature variations. The design is validated through both BSI AIS 31 and NIST tests. Specifically, the NIST SP 800-90B tests assess a min-entropy per bit of 0.995966 (Artix-7 implementation). However, as in the case of [5], the NIST SP 800-90B restart test and NIST SP 800-22 tests do not fully comply with NIST specifications. Furthermore, the code proposed on GitHub is only suitable for an FPGA implementation. An oscillation collapse-based TRNG with an odd number of inverter stages is presented in [26], achieving high randomness and resilience against PVT variations. Implemented in a 40 nm CMOS process, the design is also proven to be resistant to injection attacks. This is verified by adding an RF signal to the supply and observing the resulting variations in entropy. Hayashi et al. [27] focus instead on different types of attack against Coherent Sampling Ring Oscillator-based (COSO) TRNGs, demonstrating that side-channel attacks can be highly effective against this type of noise source. In particular, magnetic field probes can be used on the output of T flip-flops to measure power excursions, allowing the attacker to predict output random numbers. The NR and R methods are presented as possible countermeasures: at the cost of a slight COSO circuit variation, the entropy source can be effectively protected. A similar analysis is conducted in [28] on Self-Timed Ring (STR) oscillators. Two main active attacks that may cause degraded performances and/or incorrect behavior are presented, supported by analog and high-level simulations. The token injection consists of modifying the number of events propagating in the STR; the delay modification attacks add delay in one or more stages of the ring. The proposed countermeasures consist of selecting a prime number of STR stages, using an intermediate ring output as a sampling clock, and monitoring the number of events.

### 2.2. RO-Based TRNG Designs

Since the aim of this paper is to provide a comprehensive TRNG architecture, relevant references that cover not only the noise source but also the entire system are now presented. The design in [17] features a Fibonacci–Galois Ring Oscillator (FiGaRO) noise source, complemented by circuitry for on-line monitoring and error detection. Aside from an XOR tree, no additional conditioning component is used. The design occupies a large area to enhance throughput. However, the resource consumption of the noise source is not distinguished from that of the rest of the circuit. The entropy and randomness assessment is solely performed using the NIST tests; the BSI suite is not applied. A. Kamadi and Z. Abbas [29] propose a random number generator composed of an RO-based noise source, enhanced by two degrees of conditioning. An initial pre-processing is based on Maximal Length Feedback Shift registers (MLFSRs), and a second post-processing is performed by a Keccak block. Although the system appears to achieve high values of throughput, it notably lacks a NIST- or BSI-based entropy assessment. Furthermore, there is no mention of health tests or runtime monitoring of the raw bitstream. An FPGA-oriented architecture, complete with control circuitry and additional post-processing, is proposed in [30]. Programmable Delay Lines (PDLs) are employed to reduce correlation among the parallel ring oscillators. A von Neumann corrector adds a layer of post-processing, reducing the bias of the output bitstream. However, it causes a significant decrease in throughput, reducing it by a factor of 4. The testing procedure is carried out for both NIST and BSI tests, but the restart experiment is not compliant with the NIST SP 800-90B test. The TRNG design presented by Chen et al. [31] utilizes a COSO-based noise source, an online entropy estimator, and a self-adjusting mechanism to ensure high security. The ASIC implementation in 130 nm technology achieves a throughput of 4.20 Mbps and demonstrate resilience against PVT variations. The design is only validated using the NIST SP 800-22 and BSI AIS 31 statistical suites, whereas NIST SP 80-90B tests are not applied.

To the best of our knowledge, this brief review highlights the variety of RO-based noise source architectures and TRNGs proposed in the literature. Notably, most works primarily focus on the noise source, neglecting online monitoring and conditioning mechanisms. Among complete system designs, some solutions achieve compact area, high throughput, and/or effective power efficiency, but lack robust testing methodologies. Conversely, others demonstrate exhaustive validation but leave room for performance improvements. Furthermore, only one of the reviewed designs proposes an open-source solution, but it is only limited to FPGA implementations. The solution proposed in this paper addresses these gaps by offering a comprehensive, flexible, and high-performance design that has been validated through rigorous testing methodologies. Moreover, although our architecture has been implemented on FPGA, it is also suitable for ASIC, maximizing the potential of its open-source nature.

## 3. Entropy and Randomness Assessment

This section provides an overview of TRNG security evaluation metrics.

The output quality of a TRNG is given by the amount of entropy it is able to provide. Different formal definitions of entropy exist; the strictest one is the **min-entropy**, defined as the greatest lower bound for the information content of potential observations of a random variable [14]. It can be expressed in bits as:

$$H_{\min}=-\log_{2}\left[max_{i}\left(p_{i}\right)\right]$$

where $p_{i}$ is the *i*th probability of a discrete distribution having *n* possible outputs with probabilities $p_{1},...,p_{n}$.

The entropy can be assessed by means of the NIST SP800-90B tests [14] and the procedure B of the BSI suite [15]. For more technical details about the entropy estimation methodologies used in these tests, refer to [14,15].

**NIST SP 800-90B.** NIST SP800-90B tests are composed of four batteries, each one providing entropy assessment of the input bitstream:

- *IID tests*/*non-IID tests*: These two batteries are mutually exclusive as they determine whether or not the source generates Independent and Identically Distributed (IID) samples. A million samples are provided in input; if just one of the IID tests is failed, the bitstream is defined as non-IID and the other battery of tests must be applied to assess the min-entropy. Otherwise, the IID tests provide the result.
- *Restart tests*: The input consists in a 1000 × 1000 matrix. Each row corresponds to a sequence of 1000 consecutive samples to be taken immediately after the restart of the noise source. The restart tests check that there are no correlations between the samples.
- *Conditioning tests*: If the random number generator design also includes a conditioning component, these tests assess the min-entropy reduction due to the deterministic post-processing.

**BSI AIS-31, procedure B.** The BSI AIS-31 suite encompasses a total of nine tests. Tests *T6* to *T8* compose the so-called Procedure B and assess the entropy of the input bitstream. The obtained min-entropy results are generally higher compared to the ones obtained by testing the same input sequence with the NIST SP 800-90B suite.

Another important procedure for validating the security of a TRNG involves analyzing the output randomness—i.e., how much the bitstream produced by the source under test differs from that of an ideal random generator (*null hypothesis*). The metrics indicating if the *null hypothesis* holds are the *p-value* and the false positive probability α (or *level of significance*). The *p-value* is the probability that an ideal random number generator would produce a less random sequence than the one under the test. Therefore, a *p-value* closer to 1 indicates a bitstream more similar to an ideal random sequence. Instead, α is the probability that a properly functioning noise source will fail the test for a specific output. For cryptographic applications, α is chosen in the range [0.001, 0.01]. If *p-value*≥α, the sequence can be considered random with a confidence of (1−α)·100%. Also in this case, the randomness can be evaluated in two ways: NIST SP800-22 statistical test suite [21] and procedure A of the BSI suite [15].

**NIST SP 800-22**. The NIST SP 800-22 statistical test suite is composed of 15 tests, each one checking a different statistical property of the bitstream, as outlined in Table 2.

Along with the *p-value*, each test also provides as output the proportion of passing sequences:

$$Proportion=\frac{N.\;sequences\;passing\;a\;test}{Tot.\;n.\;tested\;sequences}$$

A test is passed if both *p-value* ≥α and *Prop.* fall into the confidence interval:

$$\hat{p}\pm 3\sqrt{\frac{\hat{p}\left(1-\hat{p}\right)}{m}}$$

with $\hat{p}=1-\alpha$, and *m* is the number of tested sequences. The value of *m* depends on the chosen significance value of α. If too few sequences are tested relative to α, no false positives may appear, thereby compromising the final results.

**BSI AIS-31, procedure A.** Procedure A of the BSI AIS-31 suite is composed of the remaining tests of the battery (tests *T0–T5*). The procedure requires executing test *T0* first, and then use the remaining bits produced by the suite as input for tests *T1–T5*.

Table 3 summarizes the purpose and characteristics of the different test suites described above.

## 4. Noise Source

The design investigated in this paper uses ROs as the basic blocks of the noise source.

The RO has an oscillation period equal to:

(1) $$T_{RO}=2N\cdot T_{delay}$$

where *N* is the (odd) number of inverters and $T_{delay}$ is the delay of a single inverter.

Ideally, each inverter introduces the same delay, resulting in a constant oscillation period. However, in the real case, physical phenomena affect each inverter differently, producing jitter events in the rising and falling edges of the output signal (i.e., power supply fluctuations, semiconductor noise, temperature variations, etc.). The jitter behaves as a random variable with a Gaussian profile. The variance $\sigma^{2}$ can be expressed as:

$$\sigma^{2}=\frac{8}{3\eta }\cdot \frac{kT}{P}\cdot \frac{V_{DD}}{V_{char}}$$

where η and *k* are constants, *T* is the temperature, *P* is the power, $V_{DD}$ is the supply voltage, and $V_{char}$ is the characteristic voltage of the device.

**Figure 1:**
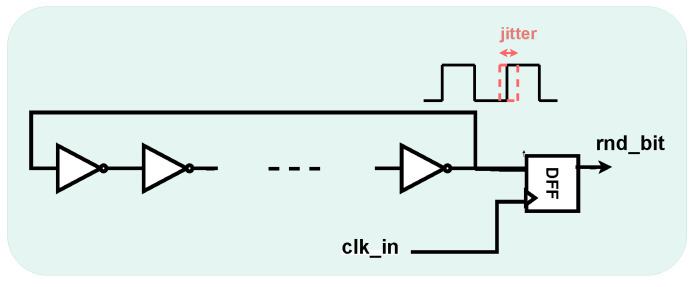
Ring Oscillator-based TRNG principle.

The jitter can be used to generate a random bit (rnd_bit) by inserting a sampler (i.e., an edge-triggered D-type Flip-Flop (DFF)) at the output of the RO, as shown in Figure 1.

The accurate mathematical analysis of jitter events in RO-based TRNGs can be found in [10,32].

The proposed TRNG uses a slightly modified version of the implementation introduced by Sunar et al. [4] and enhanced in [20]. The structure is composed of parallel ROs, whose outputs are sampled by D-type Flip-Flops (DFFs) and XOR-ed together.

**Figure 2:**
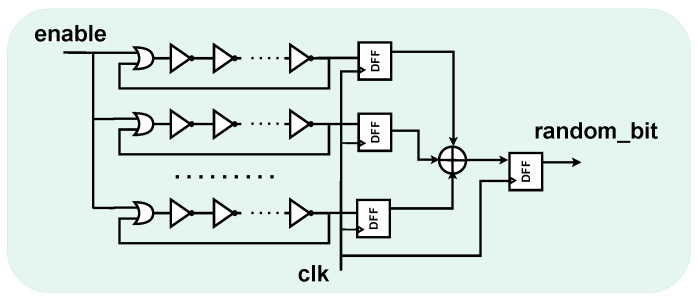
Noise source schematic.

The final random bit is the output of one last DFF collecting the XOR output. In our case, an OR gate is also inserted to connect the *enable* signal to start the oscillation, as depicted in Figure 2. For more details on the entropy justification of the source, refer to [4].

Both the number of parallel ROs and the number of inverters in each RO are key design parameters. These choices influence the Power, Performance, and Area (PPA) characteristics, as well as the statistical quality of the output bitstream. Therefore, a preliminary design space exploration is performed in Section 4.1 to determine the optimal trade-off between these factors.

### 4.1. Design Parameters Analysis

In this section, a thorough design exploration of the TRNG noise source is conducted. The primary objective is to assess the optimal configuration of the number of inverters per Ring Oscillator (RO) and the number of parallel ROs, which yields the best trade-off between area, power, and entropy of output bitstream entropy. First is the analysis of the impact on power and area, followed by the entropy assessment.

#### 4.1.1. Power and Area Analysis

**Figure 3:**
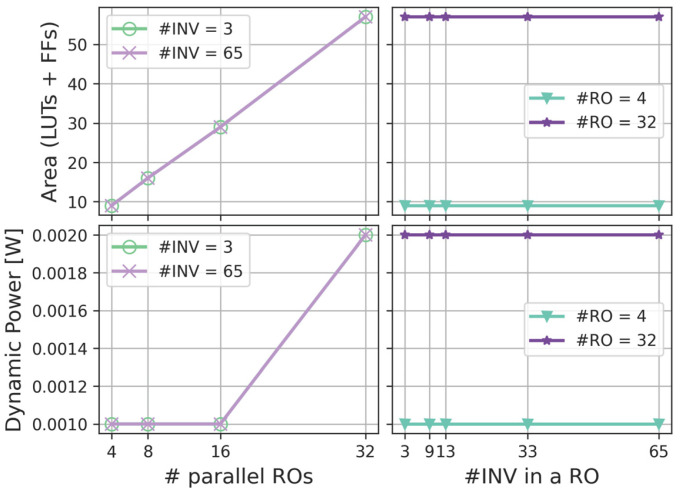
FPGA area and power results of the noise source (Artix 7).

Figure 3 depicts the area occupation and power consumption of the AMD Xilinx Artix-7 FPGA implementation (specific device: xc7a100tftg256-2) in AMD Vivado (temperature of 25 °C, operating voltage of 1 V). The clock is set to 6.7 ns in order to obtain a frequency of 150 MHz. This frequency is also the sampling frequency used to acquire all data from the real device. The implementation flow is repeated in two stages: first, by fixing the number of inverters in each RO and varying the number of parallel ROs (left-side plots), then by fixing the number of ROs and varying the number of inverters (right-side plots). The least resource-intensive configuration uses 4 ROs with 3 inverters each, while the most resource-demanding setup consists of 32 ROs, each containing 65 inverters. The results show that the number of required Look-Up Tables (LUTs), Flip-Flops (FFs), and the total power draw depend solely on the number of parallel ROs. Specifically, resource utilization ranges from 4 LUTs and 5 FFs up to 24 LUTs and 33 FFs, and dynamic power consumption spans from below 0.001 W to 0.002 W.

#### 4.1.2. Entropy Analysis

Once the design parameters are established, the variation in entropy and the randomness resulting from the different configurations can be evaluated. This examination consists of two steps: first, the analysis is conducted through simulation; then, the same evaluation is carried out on a real system, by implementing the noise source on a Xilinix Artix-7 FPGA.

**Simulation.** For the simulation, the model used to generate the delays to assign to the inverters is the one proposed by Valtchanov et al. [32]. In this model, the delay of a logic gate is expressed as the sum of multiple contributions, produced by both local and global jitter sources. In our case, only the source of true randomness, i.e., the local Gaussian jitter, is considered. Each inverter delay is composed of a superimposition of a mean value in the range [275, 282] ps (typical values [33]) and a Gaussian component of μ=0 and σ=30 ps. The inverters’ delay for each noise source configuration is generated by means of a Python script. In the testbench, each value is then assigned to one of the inverters.

**Figure 4:**
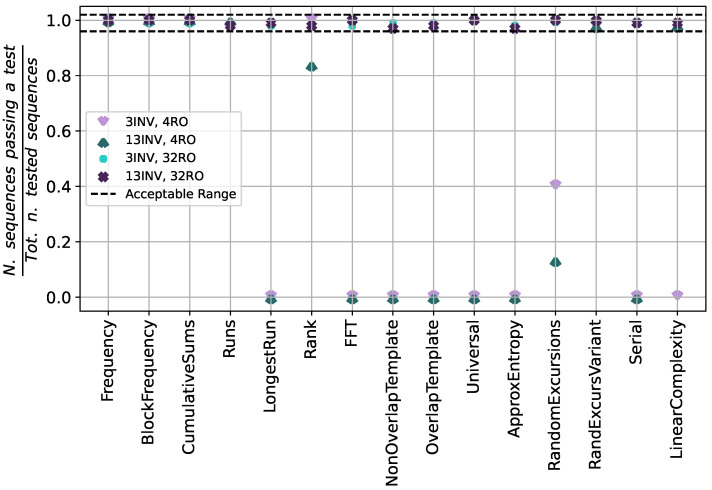
Proportions of sequences passing the 15 tests of the NIST SP 800-22 statistical suite for different design parameters (Simulation).

The sampling frequency is set initially to 500 MHz. The output bitstreams are examined using the NIST SP 800-22 [21] and the NIST SP 800-90B [14] tests. Figure 4 presents the outcomes of the NIST SP 800-22 statistical test suite (α=0.01). In the initial evaluation, 100 sequences of 1 million bits each are analyzed for each noise source configuration. The ticks on the x-axis correspond to one of the 15 tests and the values on the y-axis show the proportion of sequences that passed the test. If at least one value falls out of the dashed acceptable range, the test is failed. For a higher readability, the upper dashed line is slightly higher than 1.0; the actual value cannot obviously exceed 1.0. For the same reason, only four configurations are shown. With 4 ROs, only a few results fall within the confidence interval. The results found show that the configuration with minimum area and power also significantly compromises randomness, rendering it unsuitable for TRNG applications. By contrast, a setup with 32 parallel ROs—each featuring either 3 or 13 inverters—yields near-100% of the tested sequences passing the respective test. The obtained data are fed to the NIST SP 800-90B tests, which assess non-IID bitstreams for all the configurations. As expected, the noise sources exploiting only 4 ROs fail the tests, providing an insufficient entropy. A slightly higher entropy ($H_{min}=0.9623$) is detected in the case of the (13 INV, 32 RO) configuration with respect to the (3 INV, 32 RO) one ($H_{min}=0.9574$).

The same tests applied to data from the same noise source configurations, but with a lower sampling frequency for the DFFs (50 MHz), show the same trend. There are slight improvements for the 4 RO cases, but they are marginal and the solution is still insufficient.

**Figure 5:**
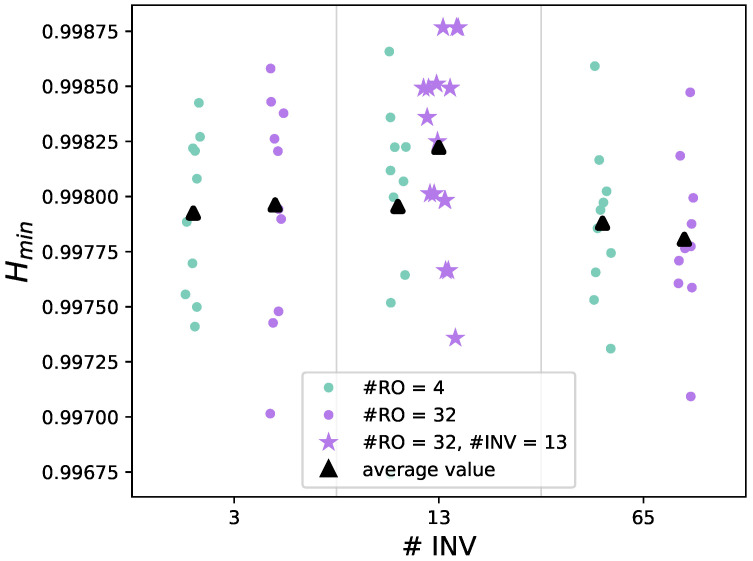
Min-entropy values from NIST SP 800-90B tests on bitstreams from various noise source configurations (Xilinx Artix-7).

**FPGA.** The noise source configurations are implemented on the Xilinx Artix-7 FPGA (specific device: xc7a100tftg256-2) on the CW305 board from NewAE’s ChipWhisperer (https://www.newae.com/products/nae-cw305). The sampling frequency is set to 150 MHz, close to the maximum limit allowed by the CW305 board (160 MHz). The operating voltage and temperature are respectively equal to 1 V and 25 °C. Unlike the simulation, all the configurations pass all 15 tests of the NIST statistical test suite. Moreover, the NIST SP 800-90B tests assess IID data. Figure 5 shows the min-entropy per-bit results of 10 different bitstreams generated by various noise source configurations. The values range from 0.9970 to 0.9988; the best ones are obtained for a (13 INV, 32 RO) configuration, yielding an average of **0.9982**.

## 5. TRNG Architecture

**Figure 6:**
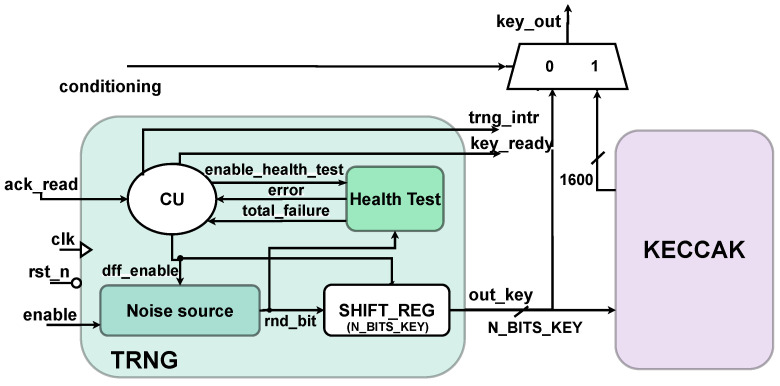
True random number generator schematic.

The architecture of the proposed True Random Number Generator (TRNG) is illustrated in Figure 6. The structure follows the NIST recommendations [14] and includes a component to perform health tests on the output of the noise source. The design is highly configurable, enabling thorough solution space exploration and allowing designers to tailor the TRNG architecture to the power, area, and randomness requirements of the target application. The configurable parameters include the parallelism of the final key, the number of Ring Oscillators (ROs) and Inverters (INVs) in the noise source, and the settings of the health tests and control unit. The parameters of these two components allow, respectively, for adjusting the stringency of tests, enabling practitioners to fine-tune the TRNG’s performance in terms of randomness and entropy based on application needs, and for setting the duration of the warm up-phase to accommodate specific environmental conditions. The following subsections explain in detail each of the different blocks of Figure 6.

### 5.1. Noise Source

Considering the analysis carried out in Section 4.1, the noise source is set to a configuration of 32 parallel ROs, each consisting of 13 inverters. These parameters give the best results in terms of entropy, with a reasonable cost in terms of area occupation (24 LUTs and 33 FFs) and power consumption (2 mW). However, in the case of specific area or power constraints, the parameterized design allows an effortless reconfiguration of a different number of ROs and inverters. The shift register collecting the serial output of the noise source is also parameterized, enabling the user to choose the output key parallelism (*N_BITS_KEY*).

### 5.2. Health Test

The *Health Test* component monitors the runtime behavior of the noise source, ensuring its correct operation. The serial random output bitstream is collected in real time for on-the-fly testing. According to NIST recommendations [14], two essential tests are required:

- **Repetition Count Test**: The purpose of this test is to verify that the data stream does not become stuck at any single value, whether 0 or 1. The steps are reported in Algorithm 1. The minimum value for the threshold *C* is obtained by the formula $C=1+\lceil \frac{-log_{2}\alpha }{H_{min}}\rceil$, with α being the false positive probability.
- **Adaptive Proportion Test**: This is a statistical procedure that examines the frequency of specific bit patterns within a sample window, aiming to identify those that occur with excessive regularity. The procedure is presented in Algorithm 2. For binary sources, the sample window, *W*, must be equal to 1024 [14], whereas the value of the threshold, *C*, depends on the desired level of entropy, as reported in [14].

If either of these tests fails, an error signal is dispatched to the Control Unit (CU), indicating the occurrence of an anomaly (*error_i*). The number of consecutive errors detected in the raw bitstream is tracked by an auxiliary counter. If this count exceeds a predefined threshold, the *total_failure* flag is asserted, signaling a critical malfunction to the CU. Consequently, the TRNG transitions into an unrecoverable state.

As previously stated, all thresholds within the *Health Test* component are user-configurable parameters that can be adjusted based on the specific application. For example, if a stringent error policy is required, the *total_failure* flag can be triggered after just three consecutive errors. Conversely, for more lenient entropy requirements, the threshold of the Adaptive Proportion test can be increased.
**Algorithm 1:** Repetition Count Test Algorithm
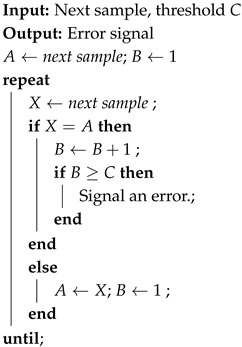


**Algorithm 2:** Adaptive Proportion Test Algorithm

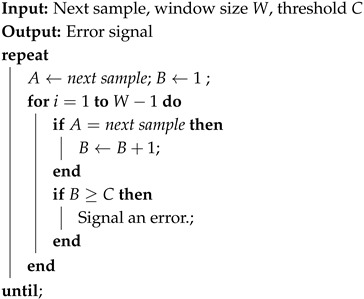



### 5.3. Control Unit

**Figure 7:**
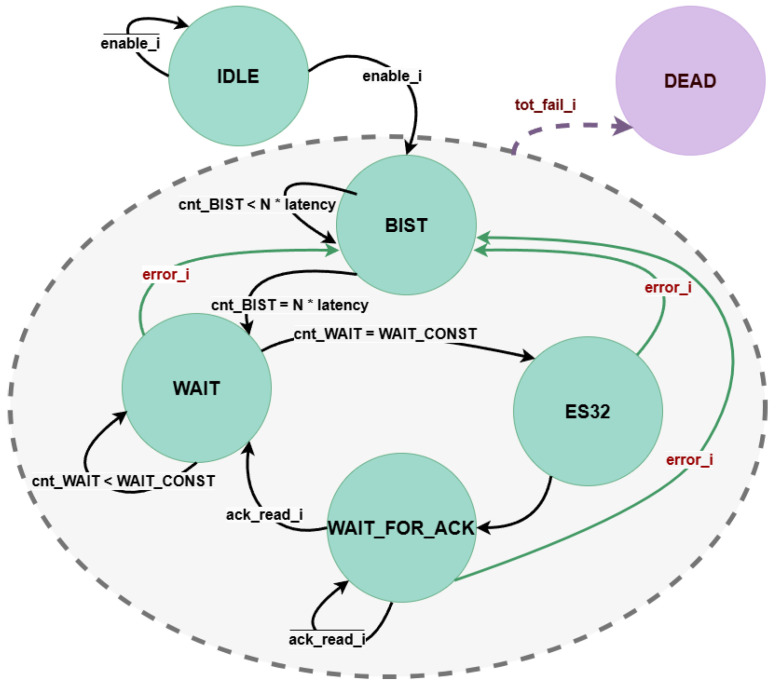
TRNG Control Unit (CU) state diagram.

The CU handles the timing and signal management of the system. The Finite State Machine (FSM) consists of six states, as represented in the state diagram of Figure 7.

Whenever the input *enable* signal is provided, the system transitions from the initial *IDLE* state to the *BIST* state. This state represents a warm-up phase; its purpose is to verify the behaviour of the noise source at system start-up: the flip-flops of the noise source and the *Health Test* component are activated (*dff_enable* and *enable_health_test* signals of Figure 6) and the serial bitstream is continuously monitored by the health tests, but no output key is generated yet. A dedicated counter (*cnt_BIST*) tracks the duration of the *BIST* state, which can be configured through the *N×latency* parameter to match the specific operating conditions of the TRNG, such as temperature and voltage. Once this period expires, the system moves to the *WAIT* state, where the output key is generated. Another configurable counter (*cnt_WAIT*) determines the duration of the state. This step may require a variable number of clock cycles depending on the selected output key parallelism. Therefore, the upper limit of the counter (*WAIT_CONST*) is parameterized so that it can be flexibly adapted consistently to the value of *N_BITS_KEY*. When the key is ready, the system transitions to the *ES32* state for one clock cycle, during which the *key_ready* flag is set and an interrupt (*trng_intr*) is generated. The TRNG remains in the *WAIT_FOR_ACK* state until an external acknowledgment of the key is received (*ack_read*). Upon receiving this acknowledgment, the system returns to the *WAIT* state to generate a new key. If an error is signaled by the *Health Test* component at any point during one of the states, the FSM transitions back to the *BIST* phase. This solution allows the noise source to recover from the temporary error without generating weak keys. If the error persists and the *total_failure* flag is received, the system is forced into the *DEAD* state, requiring a reboot of the TRNG.

### 5.4. Keccak

The design includes an additional conditioning component implementing the Keccak cryptographic function. The main objective is to increase the output entropy rate and/or obtain a more uniform distribution of the bitstream, reducing any bias introduced by the noise source [14]. Unlike health tests, which detect noise source runtime errors, the conditioning directly modifies the noise source raw bits by adding another level of processing. However, due to the deterministic nature of this function, the output entropy is inevitably reduced.

Block cipher-based and hash-based cryptographic functions are exploited as conditioning blocks. Among the suitable primitives for this task—SHA-3, AES, and ChaCha20—SHA-3 stands out as the most energy-efficient choice, consisting only of bitwise operations and generating the highest number of pseudo-random bits per round [34].

The SHA-3 standard includes cryptographic hash functions such as SHA3-224, SHA3-256, SHA3-384, and SHA3-512, as well as Extendable-Output Functions (XOFs) like SHAKE128 and SHAKE256. The Keccak algorithm is characterized by its innovative structure and robust security features, characterized by a 1600-bit state comprised of a 5 × 5 matrix of 64-bit words. Its strength is derived from the intricate transformations executed through 24 rounds of processing ($n_{r}=24$), where each round consists of five distinct steps: θ, ρ, π, χ, and ι [19]. The Keccak permutation is shown in Algorithm 3, and the implementation follows the design of [35].
**Algorithm 3:** Keccak Permutation
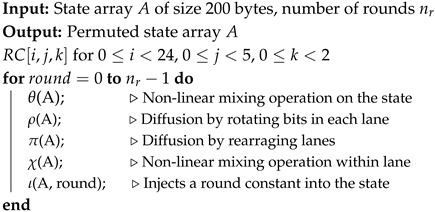


As depicted in Figure 6, the *conditioning* input allows users to choose between utilizing the TRNG alone or incorporating Keccak conditioning. This feature may be particularly useful when integrating this architecture into a larger cryptographic system, which may also require a Keccak core to implement SHA-3 functions. When *conditioning* is set to 0, both blocks operate independently and the Keccak block can be utilized for other applications. If the *conditioning* signal is set to 1, the output of the TRNG (*out_key*) is routed to the Keccak unit, which pads and processes the input message to match its 1600-bit state size. Since the parallelism of the key generated by the TRNG can be less than 1600, the system throughput can be enhanced. Nonetheless, it is crucial to maintain a balance between input size and randomness of the final keys, to avoid compromising the overall security of the system. The *key_ready* flag serves as the start pulse for the conditioning process. After 24 cycles, the final key is the 1600-bit Keccak output. The system’s interrupt and its *key_ready* flag are connected to the corresponding outputs of the Keccak component.

The Keccak block can also function as a Deterministic Random Bit Generator (DRBG), seeded by the TRNG. In this configuration, the system complies with the Random Bit Generator (RBG) class RBG2P [13], which consists of a class RBG3 (TRNG) and a DRBG (Keccak). The entropy requirements on the RBG’s final output are less strict, hence the TRNG seed parallelism can be further reduced to enhance the throughput of the system. For a DRBG with a security level of *s* bits, only s+64 bits of fresh entropy are needed [13]. For example, when using the Keccak accelerator for the SHA3-512 function (security level s=256 bits), a 320-bit seed can be generated by the TRNG block. After 24 cycles, the final 1600-bit key is produced with a throughput increase from 1 bit/cycle to 4.65 bits/cycle.

The advantages introduced by the Keccak unit are summarized in Table 4.

**Table 4:** Summary of the advantages provided by the Keccak unit.

	Output Key Characteristics	Throughput and Output Entropy Trade-Off Tuning	Flexibility
**Advantages**	More uniform distribution of output bits and reduced bias (see Table 5)	Input can be less than 1600 bits, fixed 1600-bit output: depending on the application constraints, the user can achieve higher throughput at the cost of lower output entropy and vice-versa.	The Keccak block can function as a standalone block, a conditioning block, or as a deterministic random bit generator (DRBG). Easy to integrate into larger cryptographic systems.

**Table 5:** NIST SP 800-22 test results with and without Keccak conditioning.

Test	No Conditioning			With Conditioning		
	***p-Value***	***Prop.***	**Res.**	***p-Value***	***Prop.***	**Res.**
Freq	0.3873	0.988	PASS	**0.8596**	0.987	PASS
BlockFreq	0.0786	0.993	PASS	**0.5811**	0.987	PASS
CSums	0.5287	0.988	PASS	**0.5625**	**0.989**	PASS
Runs	0.7887	0.986	PASS	0.7096	**0.992**	PASS
Long.Run	0.6470	0.990	PASS	0.1007	**0.995**	PASS
Rank	0.9061	0.988	PASS	**0.9703**	0.994	PASS
FFT	0.3686	0.989	PASS	0.1223	**0.993**	PASS
NonOvTem	0.4810	0.990	PASS	**0.5134**	0.990	PASS
OvTemp	0.7811	0.980	PASS	0.6018	**0.986**	PASS
Univ	0.1816	0.985	PASS	**0.3086**	**0.988**	PASS
ApproxEnt	0.2296	0.990	PASS	**0.2689**	0.988	PASS
RndEx	0.4826	0.989	PASS	**0.5030**	**0.990**	PASS
RndExVar	0.5091	0.989	PASS	0.4975	0.989	PASS
Serial	0.9607	0.991	PASS	0.2336	0.988	PASS
LinComp	0.5463	0.991	PASS	**0.5483**	**0.995**	PASS

## 6. Results

This section presents the results of this study. Firstly, the security of the design is analyzed in both standard (Section 6.1) and different (Section 6.2) operating conditions. Secondly, the area, power, and throughput results are evaluated and compared with existing state-of-the-art works (Section 6.3). As in Section 4.1, the TRNG is implemented on a Xilinx Artix-7 FPGA on the CW305 board. Apart from the experiments of Section 6.2, all the analyses are carried out at 25 °C, with an operating voltage of 1 V and a sampling frequency of 150 MHz.

### 6.1. Randomness Evaluation

The test suites presented in Section 3 are applied in the following subsections to evaluate the entropy and randomness of the proposed architecture.

#### 6.1.1. NIST SP 800-22

As detailed in Section 3, each one of the 15 tests of the NIST SP 800-22 statistical test suite is passed if *p-value* ≥α and the proportion of sequences passing a test (*Prop.*) falls into the confidence interval.

The 1000 sequences of $10^{6}$ bits each are collected both directly from the noise source and after processing through the Keccak block (1500-bit input from TRNG). With α=0.01, the passing rate should be ≥0.98. Table 5 reports the results, with arithmetic means provided for tests with multiple outputs. All tests are passed, with the results showing an improvement due to Keccak conditioning highlighted in bold. Specifically, the *p-values* increase for most tests, with significant improvements in cases like the Frequency or Block Frequency tests. Nevertheless, in some cases there is a slight decrease, such as the Cumulative Sums or FFT tests. This is expected since each test examines a different statistical property of the bitstream.

#### 6.1.2. NIST SP 800-90B

NIST SP 800-90B min-entropy results for the raw data can be observed in Figure 5 for the (13 INV, 32 RO) case. All IID tests are passed, with an average min-entropy of **0.9982** per bit, as reported in Table 6. For the restart tests, a sequence of 1000 consecutive samples is saved after each one of the 1000 restart procedures, which consists of the reprogramming of the CW305 board. All the tests are passed, and the resulting entropy is equal to 0.9936.

As introduced in Section 3, the conditioning tests estimate the entropy reduction caused by the additional conditioning. In the proposed design, this reduction is proportional to the increase of throughput, as explained in Section 5.4. The employment of these tests enables the calculation of the requisite key parallelism for seeding the Keccak block, thereby ensuring that it meets the specified entropy threshold. For instance, a 1206-bit key suffices to achieve a min-entropy of 0.75 [36]. After 24 cycles, the final key parallelism remains 1600 bits, increasing throughput from 1 bit/cycle to 1.3 bits/cycle.

#### 6.1.3. BSI AIS-31 Tests

Over 100 million raw bits are tested using both Procedure A (tests *T0* to *T5*) and Procedure B (tests *T6* to *T8*). Table 7 reports the average results obtained from testing multiple bitstreams. All tests are successful, and the average entropy per bit is equal to **0.9998**.

### 6.2. Different Operating Conditions

**Figure 8:**
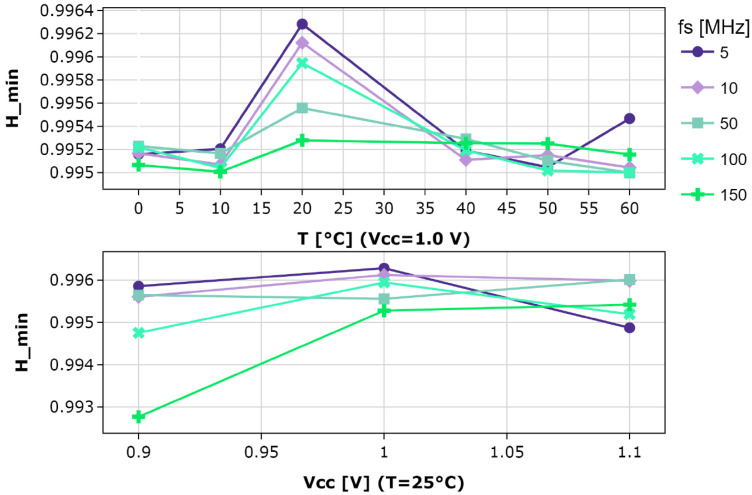
Min-entropy results with varying operating conditions.

The robustness of the proposed TRNG is tested under different operating conditions. The temperature is varied from 0 °C to 60 °C by means of a climate chamber, whereas the operating voltage, ranging from 0.9 V to 1.1 V, is directly modified through the API provided by the CW305 board. The resulting trend of the min-entropy per bit for different sampling frequencies is depicted in Figure 8.

The highest entropy is achieved at 20 °C across all sampling frequencies. The *H_min* varies by approximately $10^{-3}$, demonstrating the resilience of the TRNG to temperature fluctuations. A similar trend can be observed with voltage supply variations, with a mild increase in the range of variation. In both cases, by decreasing the sampling frequency, slightly better results of entropy are achieved. Nevertheless, the TRNG can be flexibly employed at different sampling frequencies and operating conditions without significant entropy losses.

### 6.3. FPGA Results and Comparison

Table 8 compares recent RO-based TRNGs. For a fair comparison, our results do not include the Keccak block (in this case, the total area is 4657 LUTs and 2555 FFs). Furthermore, as in most works, the power results are not mentioned; Table 8 does not comprise this metric. It is still worth addressing the fact that our implementation presents a dynamic power consumption of 0.023 W (0.547 W if also considering the Keccak accelerator).

P. Nannipieri et al. [17] implement a TRNG based on Fibonacci–Galois Ring Oscillators (FiGaRO). At the cost of four parallel FiGaRO stages, the throughput of 4 bits/cycle is achieved. Della Sala et al. [23] present a high-entropy Latched Ring Oscillator (LRO) noise source, characterized by its compact area, though with limited throughput. In [30], programmable delay inverters are exploited to tune each RO’s period accurately. The solution allows excellent control of the entropy level of the noise source but results in extensive area requirements. The Von Neumann post-processing reduces the throughput of the system. Z. Lu et al. [5] propose a low-power, low-area design with four parallel ROs and a multiphase sampler. However, some testing methods do not meet NIST or BSI standards, and there is no mention of the control circuitry or the health tests. In [22], a configurable TERO-based TRNG achieves up to 2 Mbps, with entropy and area following the state-of-the-art. No information on the used operating frequency is provided. Mentens et al. [37] introduce an architecture based on edge sampling, similar to what is done in [5], with the most significant strength being the low area occupation.

As shown in the comparison table, our design achieves higher entropy than all other references, except [23]. Moreover, it is the only design validated by both the NIST and BSI test suites, underscoring the thoroughness of its evaluation. Although the design in [23] provides the same min-entropy per bit and occupies a smaller area, it achieves significantly lower throughput than our architecture. To provide a comprehensive evaluation of area and throughput efficiency, the figure of merit *Throughput/LUT* is reported. Additionally, we include the entropy rate and *Entropy Rate/LUT* metrics to decouple the throughput results from the operating frequency, as our throughput is constrained by the maximum operating frequency of the CW305 board. When considering only the partial area, our design significantly outperforms [22,23,37]. The lower results with respect to [5] are due to the reduced operating frequency of our implementation; however, the entropy rate remains comparable, and our design achieves a higher min-entropy. For the complete architecture, our design surpasses [30] by a significant margin and exhibits a slightly lower entropy rate than [17], while achieving a higher min-entropy.

## 7. Conclusions

This work presents a highly parameterized TRNG design based on ring oscillators. Implemented on a Xilinx Artix-7 FPGA, it demonstrates strong area and power efficiency, alongside high per-bit min-entropy. The design methodology began with an in-depth analysis of the impact of key parameters on the RO-based noise source and concluded with rigorous validation using both the NIST and BSI test suites. The final system includes real-time monitoring of the raw bitstream’s quality and incorporates a Keccak accelerator, which can operate as either a conditioning block or a deterministic random bit generator. This approach enhances throughput. Furthermore, the ability to independently use both the raw bitstream and the Keccak block enables seamless integration into broader cryptographic systems—a common case for TRNGs. The ease of integration is further bolstered by the open-source nature of the proposed design. Moreover, the design flexibility makes it particularly well suited for modern cryptographic applications, as the system can be tailored to meet specific constraints on area, power, entropy, throughput, and key parallelism. For instance, in post-quantum applications, requiring large and robust keys, key parallelism can be increased and system performance can be boosted. Conversely, when using the TRNG for masking purposes, a slight decrease in entropy may be acceptable in exchange for higher throughput. Experimental results show that the TRNG achieves a per-bit min-entropy of 0.9982 (NIST) and 0.9998 (BSI) while remaining resilient to variations in temperature and supply voltage, thus mitigating invasive side-channel attacks. Future research directions include evaluating the proposed TRNG in alternative technologies, such as ASICs, and integrating it into larger cryptographic frameworks—like a crypto core—to explore potential performance gains in complex cryptographic algorithms. Furthermore, resilience against frequency injection attacks can also be examined.

## Figures and Tables

**Table 1:** Summary of the most used noise sources in TRNGs.

Ref.	Noise Source Basic Block	Key Principle
[4]	Ring Oscillator	Variable period of RO-generated clock due to physical phenomena
[16]	Metastable Ring Oscillator	Metastability of inverters
[6]	Transition Effect Ring Oscillator	Oscillatory metastability of latches
[17]	Fibonacci and Galois Ring Oscillator	Flip-flops of Fibonacci and Galois Linear Feedback Shift Registers replaced by inverters
[7]	Phase Locked Loop (PLL)	Same principle of ROs; the clock is generated by a PLL
[18]	Digital Clock Manager (DCM)	Same principle of ROs; the clock is generated by a DCM

**Table 2:** NIST SP 800-22 tests overview.

Type of Test	Description
**Frequency**	Checks for equal proportion of 1s and 0s in the entire sequence.
**Block Frequency**	Checks for equal proportion of 1s and 0s in *M*-bit blocks.
**Cumulative Sums**	Evaluates the maximal excursion of the cumulative sum random walk, where the sequence digits are adjusted (0 → −1).
**Runs**	Counts the total number of uninterrupted sequences of identical bits (runs).
**Longest Run**	Checks for the longest run of 1s in *M*-bit blocks.
**Rank**	Analyzes the rank of disjoint sub-matrices within the sequence.
**FFT**	Detects peaks in the Discrete Fourier Transform of the sequence.
**Non Overlapping Template**	Counts occurrences of *m*-bit target strings.
**Overlapping Template**	Similar to the non-overlapping template test, but considers sliding *m*-bit strings with a 1-bit overlap.
**Universal**	Measures the distance between matching patterns in the sequence.
**Approximate Entropy**	Evaluates the frequency of all possible overlapping *m*-bit patterns in the sequence.
**Random Excursions**	Counts the number of cycles with exactly *K* visits in a cumulative sum random walk.
**Random Excursions Variant**	Tracks the number of visits to a specific state in a cumulative sum random walk.
**Serial**	Measures the frequency of all possible overlapping *m*-bit patterns in the sequence.
**Linear Complexity**	Determines the length of the Linear Feedback Shift Register (LFSR) that generates *M*-bit blocks.

**Table 3:** Recap of NIST SP 800-22, NIST SP 800-90B, and BSI AIS-31 tests for TRNG validation.

Test Suite	NIST SP 800-22	NIST SP 800-90B	BSI AIS-31	
			Procedure A	Procedure B
**Focus**	Randomness assessment	Entropy assessment	Randomness assessment	Entropy assessment
**Tests**	15 statistical tests	IID tests, non-IID tests, restart tests, conditioning tests	*T0*, *T1* to *T5*	*T6* to *T8*

**Table 6:** NIST SP 800-90B IID test results.

Category	Test	C[i][0]	C[i][1]	C[i][2]
Statistical Tests	Excursion	9	0	6
	NumDirectionalRuns	34	0	6
	LenDirectionalRuns	4	2	5
	NumIncreasesDecreases	28	0	6
	NumRunsMedian	6	0	7
	LenRunsMedian	3	3	8
	AvgCollision	6	0	167
	MaxCollision	7	1	5
Periodicity Tests	Periodicity (1)	6	0	13
	Periodicity (2)	6	0	10
	Periodicity (8)	39	1	5
	Periodicity (16)	6	0	14
	Periodicity (32)	6	0	35
Covariance Tests	Covariance (1)	6	0	9
	Covariance (2)	6	0	15
	Covariance (8)	6	0	6
	Covariance (16)	13	0	6
	Covariance (32)	6	0	23
Compression Test	Compression	21	0	6
Chi Square Tests	Chi Square independence	p_*value* = 0.6724		
	Chi Square goodness of fit	p_*value* = 0.3689		
LRS		46		
**Min-Entropy**		**0.9982**		

**Table 7:** BSI AIS-31 test results.

Procedure	Test	Pass Rate
Procedure A	*T0*—Disjointness	100%
	*T1*—Monobit	100%
	*T2*—Poker	100%
	*T3*—Runs	100%
	*T4*—Long Run	100%
	*T5*—Autocorrelation	100%
Procedure B	*T6a*—Uniform Distribution	100%
	*T6b*—Uniform Distribution	100%
	*T7a*—Multinomial Distribution	100%
	*T7b*—Multinomial Distribution	100%
	*T8*—**Byte Entropy Estimation:** **7.998217**	

**Table 8:** Comparison of different TRNG implementations.

Ref.	FPGA Device	Area		Thr. [MBps]	Freq. [MHz]	Entropy Rate [Bits/Cycle]	Thr./LUTs	Entr.Rate/LUTs	Min-Entropy	
		LUTs	FFs						NIST	BSI
[17]	Stratix IV	288	190	400	100	4.00	1.39	0.0139	0.995	-
[23]	Spartan 6	4 †	3 †	0.76	50	0.0152	0.19 †	0.0038 †	-	0.9998
[30]	Spartan 3A	528	177	6	24	0.25	0.011	0.0005	-	0.9993
[5]	Artix 7	24 †	33 †	275.8	275.8	1.00	11.49 †	0.0417 †	0.9973	-
[22]	Artix 7	40 ‡	29 ‡	1.91	-	-	0.048 ‡	-	-	0.9993
[37]	Spartan 6	16 ‡	11 ‡	1.15	100	0.0115	0.072 ‡	0.0007 ‡	0.910	-
**OURS**	**Artix 7**	**98**	**194**	**150**	**150**	**1.00**	**1.53**	**0.0102**	**0.9982**	**0.9998**
		**24 †**	**33 †**				**6.25 †**	**0.0417 †**		

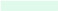
† only noise source area. 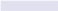
‡ noise source + control logic area

## Data Availability

Data are contained within the article.

## Abbreviations

The following abbreviations are used in this manuscript:

ASIC	Application-Specific Integrated Circuit
BSI	Bundesamt für Sicherheit in der Informationstechnik
DCM	Digital Clock Manager
DRBG	Deterministic Random Bit Generator
FPGA	Field Programmable Gate Array
PLL	Phase Locked Loops
PPA	Power, Performance, and Area
PRNG	Pseudo Random Number Generators
RO	Ring Oscillators
NIST	National Institute of Standards and Technology
TRNG	True Random Number Generator
